# The Interplay of Stress and Sleep Impacts BDNF Level

**DOI:** 10.1371/journal.pone.0076050

**Published:** 2013-10-16

**Authors:** Maria Giese, Eva Unternaehrer, Serge Brand, Pasquale Calabrese, Edith Holsboer-Trachsler, Anne Eckert

**Affiliations:** 1 Neurobiology Laboratory for Brain Aging and Mental Health, Psychiatric University Clinics, University of Basel, Basel, Switzerland; 2 Division of Molecular and Cognitive Neuroscience, University of Basel, Basel, Switzerland; 3 Department of Clinical Psychology and Epidemiology, University of Basel, Basel, Switzerland; 4 Depression and Sleep Research Unit, Psychiatric University Clinics, University of Basel, Basel, Switzerland; 5 Transfaculty Research Platform, Molecular and Cognitive Neuroscience, Univ. of Basel, Basel, Switzerland; Chiba University Center for Forensic Mental Health, Japan

## Abstract

**Background:**

Sleep plays a pivotal role in normal biological functions. Sleep loss results in higher stress vulnerability and is often found in mental disorders. There is evidence that brain-derived neurotrophic factor (BDNF) could be a central player in this relationship. Recently, we could demonstrate that subjects suffering from current symptoms of insomnia exhibited significantly decreased serum BDNF levels compared with sleep-healthy controls. In accordance with the paradigm indicating a link between sleep and BDNF, we aimed to investigate if the stress system influences the association between sleep and BDNF.

**Methodology/Principal Findings:**

Participants with current symptoms of insomnia plus a former diagnosis of Restless Legs Syndrome (RLS) and/or Periodic Limb Movement (PLM) and sleep healthy controls were included in the study. They completed questionnaires on sleep (ISI, *Insomnia Severity Index*) and stress (PSS, *Perceived Stress Scale)* and provided a blood sample for determination of serum BDNF. We found a significant interaction between stress and insomnia with an impact on serum BDNF levels. Moreover, insomnia severity groups and score on the PSS each revealed a significant main effect on serum BDNF levels. Insomnia severity was associated with increased stress experience affecting serum BDNF levels. Of note, the association between stress and BDNF was only observed in subjects without insomnia. Using a mediation model, sleep was revealed as a mediator of the association between stress experience and serum BDNF levels.

**Conclusions:**

This is the first study to show that the interplay between stress and sleep impacts BDNF levels, suggesting an important role of this relationship in the pathogenesis of stress-associated mental disorders. Hence, we suggest sleep as a key mediator at the connection between stress and BDNF. Whether sleep is maintained or disturbed might explain why some individuals are able to handle a certain stress load while others develop a mental disorder.

## Introduction

Sleep is associated with physical and mental health [1], [2], [3], [4]. Sleep loss impairs various endocrine, physiological [5] as well as neuronal functions [6], [7], [8] and is often followed by higher stress vulnerability, reduced environmental adaptation and cognitive impairment [9]. Moreover, insomnia is often observed in many stress-related disorders [3]. Evidence indicates that BDNF could play a role in this association: i) in animal studies BDNF levels decreased after chronic stress [10], [11], [12], [13], ii) serum BDNF levels decreased in stress-related major depressive disorder [14], [15], [16], iii) BDNF plays a role in sleep homeostasis [17], [18], [19]. Additionally, a recent study by our group showed that insomnia is associated with decreased serum BDNF levels [20].

The major stress response system, the hypothalamic–pituitary–adrenal (HPA) axis, facilitates the adaptation to stress. Chronic stress can lead to a deregulation in this biological stress system [21], [22], [23], [24], [25], which was suggested to influence BDNF levels in limbic brain structures in animal studies as well as in human blood compartments, namely serum and plasma [10], [11], [12], [14], [15], [16]. In rodents it was shown that acute and chronic stress decreased levels of BDNF in the dentate gyrus and the hippocampus. This reduction seemed to be mediated partly via stress-induced glucocorticoids [26].

Next to the nervous system, BDNF is found in the periphery of humans and other mammals [27], [28]. Since the protein can cross the blood brain-barrier in both directions, circulating BDNF correlates with cortical BDNF concentrations [29]. To what extent peripheral BDNF levels correspond to brain BDNF levels remains unknown. However, the use of serum BDNF concentration as potential indicator of brain alteration is justified by extensive evidence [30].

In addition to the well-known functions of BDNF acting as a neurotrophin, several studies involving human subjects and animal models provide preliminary data supporting a role for BDNF also in stress and mood disorders [14], [31], [32], [33], [34], [35].

Altogether, these findings suggest a possible role of the interplay between stress, sleep and BDNF, however, the relation remains unclear. Therefore the aim of this study was to test how stress and sleep could affect serum BDNF levels. To elicit our hypothesis, participants with current symptoms of insomnia and non-sleep disturbed controls were asked to complete questionnaires on insomnia and stress experience. In the same sample, we could already demonstrate an association between decreased serum BDNF levels and insomnia severity [20].

## Materials and Methods

### Participants and procedure

Participants were recruited in three different ways. First, patients with a former diagnosis of Restless Legs Syndrome (RLS) and/or Periodic Limb Movement (PLM) were recruited from the Department of Sleep and Depression Research of the Psychiatric University Clinic Basel, Switzerland. These patients are expected to suffer from secondary sleep disturbances, due to obvious symptom experience and not due to other physical and mental disorders. Second, we recruited participants using a *study participant database* provided by the Department of Psychology of the University of Basel and third – in order to reach older participants – we asked members of an organization of the elderly to participate.

At first contact, the study was explained by phone, e-mail or face-to-face. When interested in participation, patients and controls were thoroughly informed about the study and received an information-package containing detailed study information and several questionnaires, which they were asked to complete at home. Participants were then invited to a personal appointment in the facilities of the Department of Sleep and Depression Research of the Psychiatric University Clinic Basel in Switzerland, where they were requested to arrive fasting in the morning at 7.45 a.m. to provide a blood sample and complete several questionnaires. The study protocol was approved by the local ethical committee of Basel (“Ethikkommission beider Basel” - EKBB).

Subjects with severe medical or neurological conditions (e.g. intoxication, drug abuse, stroke), lifetime diagnosis of alcohol dependence and diagnosis of current major depression were not included in the study. All participants gave written informed consent in accordance with the declaration of Helsinki and received financial compensation. A total of 50 participants were included in the study (for further details see Table 1 – characteristics of study participants). Besides a sociodemographic questionnaire to assess sex, age and BMI, participants also had to indicate substance consumption (coffee, cigarettes and alcohol) and medication intake, which all could interfere with the biological analyzes [36], [37], [38], [39]. These potential confounders were statistically controlled and only smoking (stratified into current smokers who smoke daily or occasionally at least on cigarette per week and non-smokers) was associated with serum BDNF levels and was therefore included in subsequent analyzes.

**Table 1:** Descriptive characteristics of study participants.

		*M*	*SD*
Age (years)		54.7	11.6
Body mass index		27.2	5.1
Serum BDNF (ng/ml)		18.01	9.84
Score on the ISI		9.4	6.6
Score on the PSS		26.5	7.4

		*N*	%
Sex	male	25	50
	female	25	50
Former diagnosis	none	24	48
	RLS/PLM	26	52
Cigarette smoking	smoker	16	32
	non-smoker	33	66
	missing	1	2

A total sample size of N = 50 was included in the analysis. Descriptive data are presented in means (M) and standard deviations (SD). Absolute numbers of participants are given (N) and expressed as percentage (%).

**Abbreviations:** means (*M*); standard deviation (*SD*), brain-derived neurotrophic factor (BDNF); Insomnia Severity Index (ISI); Perceived Stress Scale (PSS); restless legs syndrome (RLS), periodic limb movement (PLM).

### BDNF analyzes

For serum sampling, blood was obtained in a serum separator tube from the antecubital vein between 7.45 and 8.00 a.m. After 30 minutes of clotting time, the whole blood was centrifuged at 1000×g for 30 minutes to separate and collect the serum. Aliquots were kept at −80°C until assaying.

Serum BDNF levels (concentration: ng/ml) were assessed with an enzyme-linked immunoabsorbent assay (ELISA) kit (Promega BDNF Emax®, Madison, Wis.). Samples were appropriately diluted (between 1:100–1:150) and detection of total soluble BDNF was carried out in an antibody sandwich format like described in the manufacturers protocol. The absorbance was measured within 30 minutes in a microplate reader at 450 nm to determine BDNF concentrations according to the standard curve. All assays were performed in duplicates and means were calculated.

### Questionnaires

#### Sleep

Participants completed the *Insomnia Severity Index (ISI)* [40], which is an established screening questionnaire for insomnia. The ISI is a 7-item scale that yields a quantitative index of insomnia severity. Participants were asked to specify on a 5-point Likert scale ranging from 0 ( =  not at all) to 4 ( =  very much) to what extend they suffered from difficulties in falling asleep or maintaining sleep and early awakenings during the last two weeks. Additionally they were asked to rate satisfaction with sleep, daytime sleepiness and worrying about poor sleep. A sum score of 8 to 14 indicates that the respondent suffers from sub threshold insomnia. A score of 15 and higher indicates clinical insomnia.

#### Stress

Stress perception was assessed using the German versions of the *Perceived Stress Scale (PSS)* [19], which consists of 10-items and is used to determine perceived overall stress occurring in the previous month. The German version of the PSS has satisfactory internal consistency and test-retest reliability [29]. Answers were given on a 5-point Likert scale ranging from 1 ( =  never) to 5 ( =  very often), with higher scores reflecting greater perceived stress.

### Statistical analysis

For descriptive purposes means and standard deviations and errors were calculated for stress, sleep and serum BDNF levels and potential confounding variables.

First, potential confounders were examined and later included in the analyses if they were significantly correlated with scores on the ISI, PSS or serum BDNF levels (Pearson's (*r_p_*) or Spearman's (*r*_s_) correlation coefficients). Second, we performed an ANCOVA to assess the main effects of sleep and stress and their interaction with regard to serum BDNF levels. Third, we conducted mediator models using the bootstrap calculation of an SPSS macro according to Preacher and Hayes [41]. All analyses were performed using SPSS Statistics 20 for Macintosh. A *p*-value smaller than 0.05 was considered as significant.

## Results

### Participant characteristics and potential confounders

Descriptive characteristics of participants are shown in Tab. 1. There was a significant association of BDNF with smoking (*r_p_* = 0.408, *p* = 0.004), which was included as a covariate in further analyses. To assess the influence of insomnia severity we divided all participants into three subgroups according to their score on the insomnia severity index: subjects with no insomnia (score 0–7, n = 24), sub threshold insomnia (score 8–14, n = 16) and clinical insomnia (score 15–30, n = 10). Patients and controls were stratified into the groups as follows: i) no insomnia: 17 with no, 7 with a former diagnosis of RLS/PLM; ii) sub threshold insomnia: 5 with no, 11 with a former diagnosis of RLS/PLM; iii) clinical insomnia: 2 with no, 8 with a former diagnosis of RLS/PLM. Serum BDNF levels did not differ between diagnosis groups (RLS, PLM, sleep-healthy) and were not influenced by medication [20].

### Serum BDNF levels, sleep and stress

**Figure 1:**
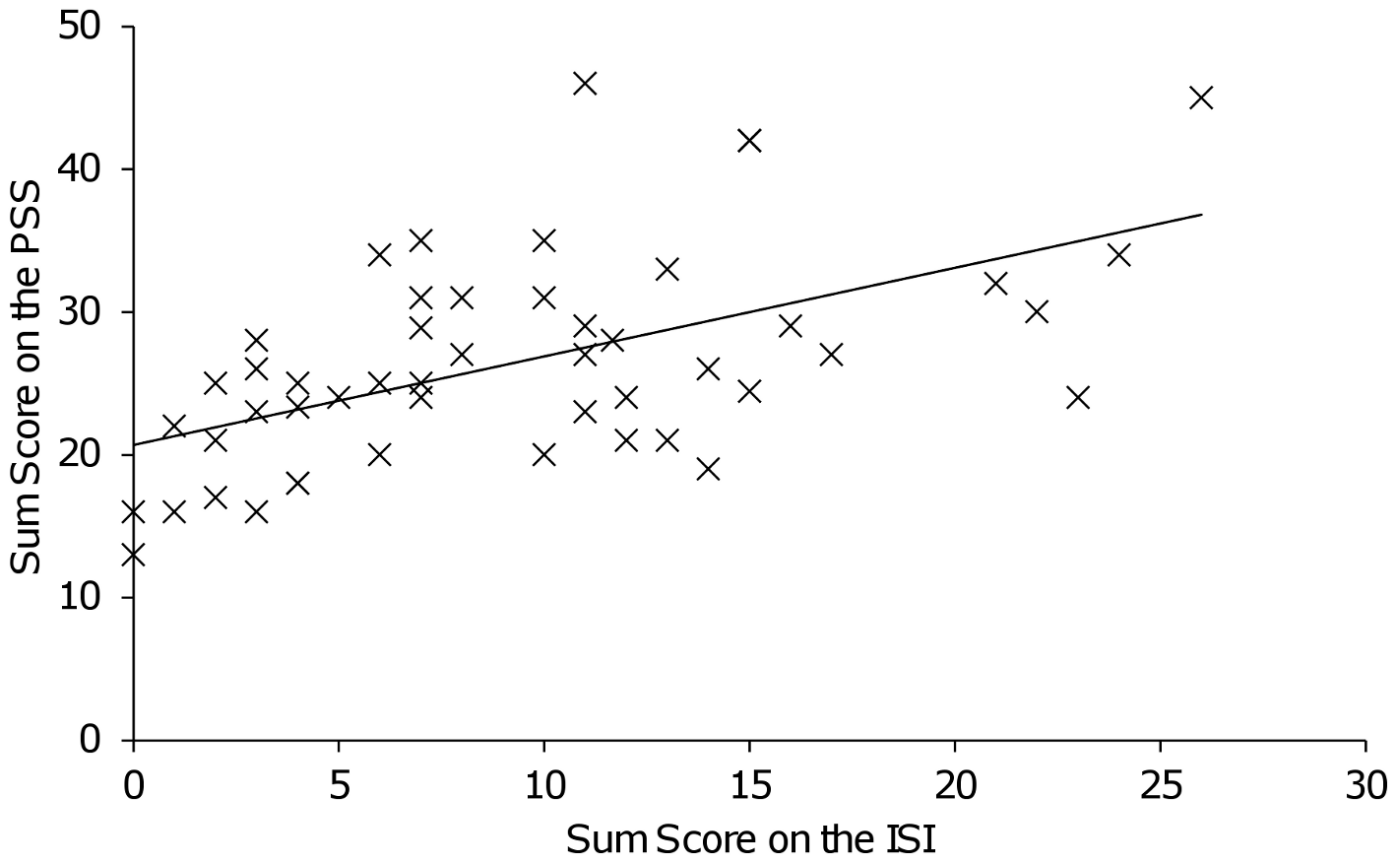
Stress experience in subjects suffering from current insomnia. Correlation between insomnia severity score (indicated by the *Insomnia Severity Index* (ISI)) and stress perception (indicated by the *Perceived Stress Scale* (PSS)). Analysis showed a significant correlation between scores on the ISI and the PSS across the whole sample (*r_p_* = 0.548, *p*<0.001). * Denotes statistical significance at *p*<0.05.

**Figure 2:**
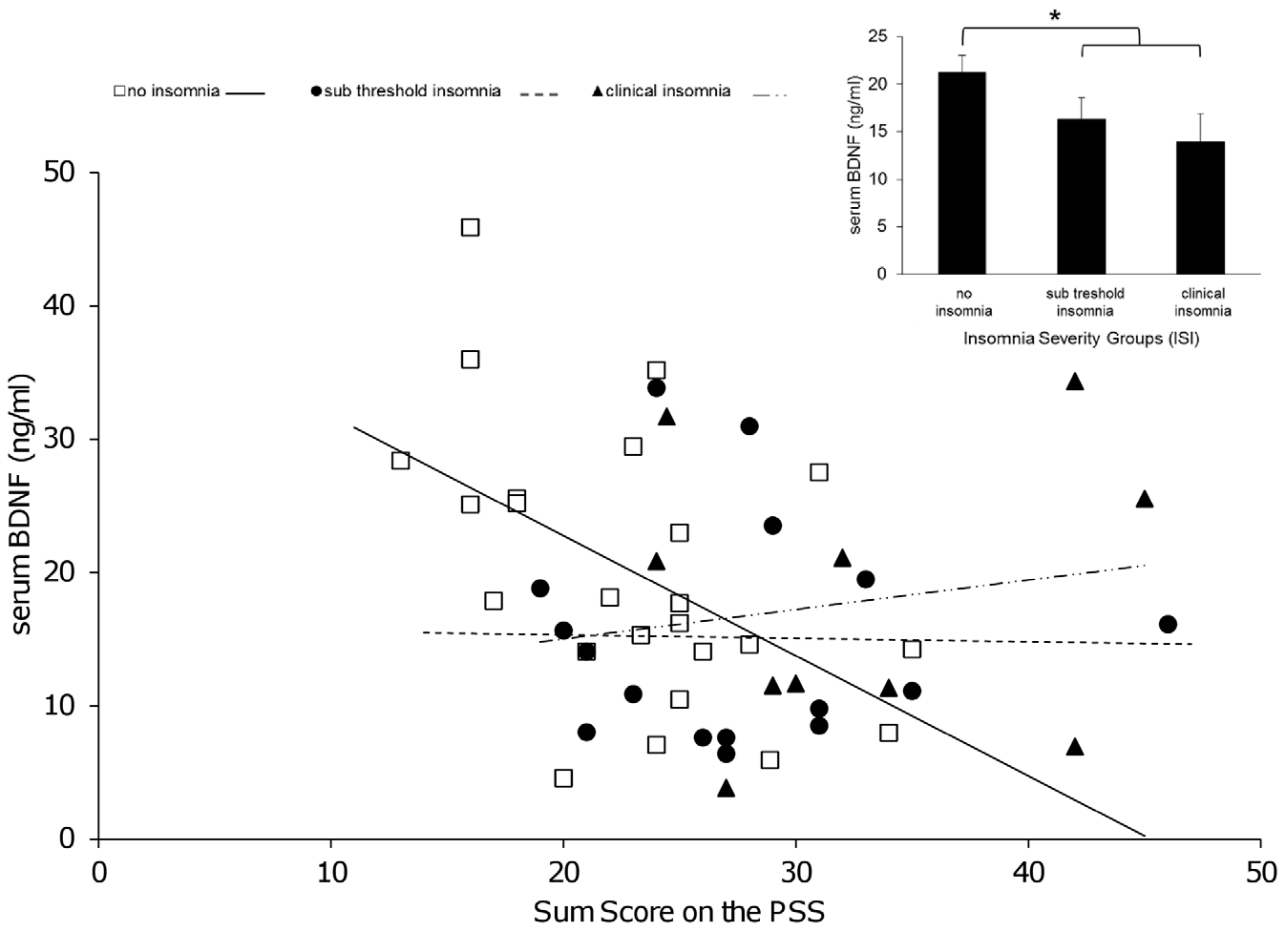
Correlation between serum BDNF levels and scores on the *Perceived Stress Scale* (PSS) by insomnia severity groups according to the *Insomnia Severity Index* (ISI). Analyses showed a significant correlation (partial correlation controlled for smoking) between BDNF and stress only in subjects with no insomnia (*r_p_* = −0.511, p = 0.013) compared to subjects with sub threshold (*r_p_* = 0.069, *p* = 0.814) or clinical insomnia (*r_p_* = 0.199, *p* = 0.608). White squares represent subjects with no insomnia, black circles represent subjects with sub threshold and black triangles represent subjects with clinical insomnia. (Inset) Mean serum BDNF levels of the insomnia severity groups. Plotted means and standard errors estimated by ANCOVA with serum BDNF as dependent variable, insomnia severity group as independent variable and smoking as covariate. For all three insomnia severity groups the overall effect on serum BDNF was not significant (*F*(2) = 2.67; *p* = 0.080). Contrasts showed that serum BDNF levels in the group with no insomnia were significantly higher compared to the groups reporting sub threshold and clinical insomnia (*F*(1) = 5.33; *p* = 0.026); (no insomnia n = 24; sub threshold insomnia n = 16, clinical insomnia n = 10). * Denotes statistical significance at *p*<0.05

As expected, stress experience on the PSS increased with rising sum score of insomnia severity (*r_p_* = 0.548; *p*<0.001) (Fig. 1). Previously, we could show that an increase in severity of insomnia was associated with a decrease in serum BDNF levels of the same sample [20]. Serum BDNF levels in the group with no insomnia were significantly higher compared to the groups reporting sub threshold and clinical insomnia (Fig. 2 inset). To further elucidate the complex interplay between stress, insomnia and BDNF we calculated an ANCOVA (BDNF as dependent variable, insomnia and stress as independent variables and smoking as co-variable). We found a significant interaction between stress and insomnia with an impact on serum BDNF levels (*F* = 6.180, *p* = 0.017). Moreover we found a significant main effect for the independent variables: (i) insomnia severity groups (*F* = 7.775, *p* = 0.008) with an incremental decrease in serum BDNF levels from the no insomnia to the sub threshold and to the clinical insomnia group, (ii) score on the PSS (*F* = 8.230, *p* = 0.006), with decreased serum BDNF levels associated with increased scores on the PSS and (iii) the covariate smoking (*F* = 14.154, *p*<0.001). Inclusion of current RLS symptoms severity, former diagnosis (RLS, PLM, control) and alcohol consumption as additional covariates did not affect the interaction and main effects of sleep and stress on serum BDNF levels. Furthermore, these additional covariates were not significantly associated with serum BDNF levels (all *p*>0.1). Of note, an association between the PSS and BDNF was only observed in subjects with no insomnia (*r_p_* = −0.511, *p* = 0.013) compared to subjects with sub threshold (*r_p_* = 0.069, *p* = 0.814) or clinical insomnia (*r_p_* = 0.199, *p* = 0.608) (Fig. 2).

**Figure 3:**
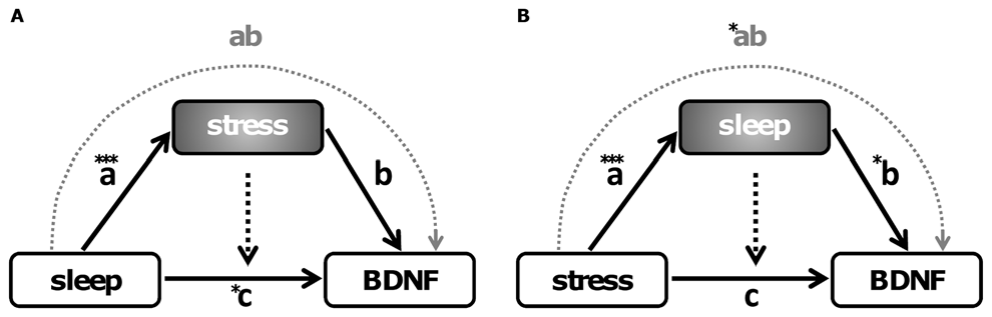
Mediation models for the interplay between stress, sleep and BDNF. (A) Stress as a mediator in the relationship between sleep and BDNF. Analyses revealed a significant ‘a’ path (*t* = 4.36; *p*<0.001) between sleep and stress. The ‘b’ path between stress and BDNF was not significant (*t* = −0.92; *p* = 0.365). The ‘c’ path between sleep and BDNF was significant after inclusion of stress as mediator (*t* = −2.05; *p* = 0.046). The indirect ‘ab’ path was not significant (bootstrap 95% confidence intervals: lower  = −0.385, upper  = 0.169, *p*>0.05). (B) Sleep as mediator in the relationship between stress and BDNF. The ‘a’ path between stress and sleep was significant (*t* = 4.36; *p*<0.001), as was the ‘b’ path between sleep and BDNF (*t* = −2.05; *p* = 0.046). The ‘c’ path between stress and BDNF was not significant when the mediator sleep was included (*t* = −0.92; *p* = 0.365). In this model the indirect ‘ab’ path was significant (bootstrap 95% confidence intervals: lower  = −0.522, upper  = −0.046; *p*<0.05). Both models explain 27.3% of the variation (adjusted R square) in serum BDNF levels (*F*(7.45) = 7.013; *p*<0.001). Smoking was included as covariate in both models. * Denotes statistical significance at *p*<0.05 and *** *p*<0.001.

To further elucidate a mutual relationship between the three parameters stress, sleep and BDNF we calculated a mediation model as described by Preacher & Hayes [41]. In the first model (Fig. 3a) we defined stress as mediator of the relationship between sleep and BDNF. Results showed a significant ‘a’ path (*t* = 4.36; *p*<0.001) between sleep and stress. The ‘b’ path between stress and BDNF was not significant (*t* = −0.92; *p* = 0.365). The ‘c’ path between sleep and BDNF was significant after inclusion of stress as mediator (*t* = −2.05; *p* = 0.046). The indirect ‘ab’ path was not significant (bootstrap 95% confidence intervals: lower  = −0.385, upper  = 0.169, *p*>0.05). In the second model (Fig. 3b) we defined sleep as mediator of the relationship between stress and BDNF. As expected, the ‘a’ path (*t* = 4.36; *p*<0.001) between stress and sleep was significant. Here, also the ‘b’ path between sleep and BDNF was significant (*t* = −2.05; *p* = 0.046). The ‘c’ path between stress and BDNF was not significant when sleep was included as a mediator (*t* = −0.92; *p* = 0.365) and this time the indirect ‘ab’ path was significant (bootstrap 95% confidence intervals: lower  = −0.522, upper  = −0.046; *p*<0.05). Both models can explain 27.3% of the variation (adjusted R square) in the serum BDNF levels (*F*(7, 45) = 7.013; *p*<0.001).

## Discussion

The aim of this study was to investigate if the influence of the association between stress and sleep has an impact on serum BDNF levels. As previously shown, insomnia was correlated with decreased serum BDNF levels [20]. In the present study we found an interaction between stress and insomnia, which affects serum BDNF levels. Based on subgroup analyses (no insomnia, sub threshold and clinical insomnia) an association between stress and BDNF was strongly pronounced in sleep healthy participants, while in subjects suffering from symptoms of insomnia this association was attenuated. Therefore the present results are in line with the assumption that stress affects sleep and BDNF levels and that sleep *vice versa* has an impact on the link between stress and BDNF.

To further elucidate this finding we investigated two mediator models (Fig. 3a and b). In the first model, stress was defined as mediator of the relationship between sleep and BDNF and in the second model sleep was determined as the mediator of the relationship between stress and BDNF. The first model did not identify stress as a mediator of the relationship between sleep and BDNF. Notably, our second model revealed that sleep mediated the association between stress and BDNF. Importantly, this means that increased stress negatively affects sleep and in turn decreases BDNF levels. Our main finding is partly in line with previous research showing a major role for stress on BDNF regulation. Our results suggest for the first time sleep as a key mediator in the connection between stress and BDNF.

The results of our study revealed that only subjects who suffer from increased stress and at the same time from comorbid sleep disturbances show decreased BDNF levels. We argue that stressed while non-sleep disturbed subjects have BDNF levels similar to non-stressed subjects. This highlights the importance of good sleep in dealing with stress. We assume that the interplay between decreased BDNF, chronic sleep impairment and increased stress levels is an essential mechanism in the pathogenesis of stress-associated mental disorders. Whether sleep is maintained or disturbed might explain why some individuals are able to handle a certain stress load while others develop a mental disorder. Thereby, adequate levels of BDNF could promote neuronal plasticity, a factor supporting mental health.

Our study is the first to combine two predictors, sleep and stress, regarding BDNF levels. Moreover, we were able to further disentangle the intricate relationship between sleep and stress. This is important since stress-related mood disorders are characterized by multifactorial features and often appear in comorbidity with sleep disorders. Additional strength is that we did not simply compare patients and controls by diagnosis but investigated insomnia severity in all subjects. Emphasized by the fact that consideration of diagnosis as covariating factor did not affect our main results; the possibility that our findings are biased by diagnosis is ruled out. Therefore we are able to make statements about the consequences of insomnia in general rather than related to a specific clinical diagnosis. This is important with regard to underlying mechanisms independent of a diagnosis. In sleep healthy subjects we noted a strong association between stress and serum BDNF levels. This could indicate that in these subjects stress results in decreased BDNF levels, while this association was diminished in sleep disturbed subjects. A reasonable argument could be that chronic sleep loss, probably caused by stress, is associated with reduced circulating serum BDNF in general, as well as an impaired stress adaptation system.

This study has also some limitations. First, there could be various other biological factors influencing BDNF levels, which were not analyzed in this study such as physical activity [42], alterations in neurotransmitter and hormone production and function, cytokines [43], as well as genetic and epigenetic mechanisms [44], [45]. Second, in this study only total serum BDNF was investigated, neglecting the individual isoforms pro- and mature BDNF. Both isoforms are considered to transmit important, opposing cellular effects via their appropriate receptors p75 neurotrophin receptor (p75^NTR^) and tropomyosin-related tyrosine kinase receptor B (TrkB). Recently, Yoshida and coworkers could demonstrate the measurement of pro- and mature BDNF from human serum of healthy subjects for the first time [46]. Therefore, it would be beneficial to especially quantify individual levels of pro- and mature BDNF to reveal if reduced levels of total serum BDNF are driven by an imbalance of either pro- or mature BDNF, which was already shown for patients suffering from major depression [47]. Subsequent studies should, next to total levels of peripheral BDNF, focus on individual isoforms, since they could unfold complementary information regarding a decrease or increase of BDNF protein and the corresponding biological mechanisms.

Third, further studies should examine other biological correlates of sleep impairment, difficulties in stress coping and elevated stress perception. Fourth, studies on patients with specific sleep-related disorders, especially primary insomnia, or with experimentally induced sleep restriction have to be conducted to generalize these results. Therefore, we suggest that future studies should investigate – next to subjective psychological stress ratings – objective endocrine stress parameters, such as cortisol and a greater sample size should be aspired. Fifth, there might be additional cofactors that were not assessed, such as co-morbidly existing somatic, e.g. pain perception, psychiatric disorders and related impairment of cognition [48] or nutritional intake.

## Conclusions

To the best of our knowledge, this is the first study to show that stress experience and subjective sleep perception interact with each other and affect BDNF levels. We suggest that this interplay is involved in the pathology of stress-associated mental disorders. We add new weight to our previous suggestion [20] to seriously consider the assessment of sleep when analyzing BDNF as a marker in stress-related mood disorders, since several studies have found decreased levels of serum BDNF in various stress-related mental disorders, such as depression [14], [49], posttraumatic stress disorder [50] and burnout syndrome [46], all of which are associated with sleep-related problems. The underlying biological mechanisms, e.g. involvement of the stress hormone system, have to be elucidated in future studies.
